# Exposure to a High-Fat Diet during Early Development Programs Behavior and Impairs the Central Serotonergic System in Juvenile Non-Human Primates

**DOI:** 10.3389/fendo.2017.00164

**Published:** 2017-07-21

**Authors:** Jacqueline R. Thompson, Jeanette C. Valleau, Ashley N. Barling, Juliana G. Franco, Madison DeCapo, Jennifer L. Bagley, Elinor L. Sullivan

**Affiliations:** ^1^Division of Neuroscience, Oregon National Primate Research Center, Beaverton, OR, United States; ^2^Division of Cardiometabolic Health, Oregon National Primate Research Center, Beaverton, OR, United States; ^3^Department of Biology, University of Portland, Portland, OR, United States

**Keywords:** maternal, high-fat diet, obesity, anxiety, stereotypy, mental health, neurodevelopmental, cortisol, serotonin

## Abstract

Perinatal exposure to maternal obesity and high-fat diet (HFD) consumption not only poses metabolic risks to offspring but also impacts brain development and mental health. Using a non-human primate model, we observed a persistent increase in anxiety in juvenile offspring exposed to a maternal HFD. Postweaning HFD consumption also increased anxiety and independently increased stereotypic behaviors. These behavioral changes were associated with modified cortisol stress response and impairments in the development of the central serotonin synthesis, with altered tryptophan hydroxylase-2 mRNA expression in the dorsal and median raphe. Postweaning HFD consumption decreased serotonergic immunoreactivity in area 10 of the prefrontal cortex. These results suggest that perinatal exposure to HFD consumption programs development of the brain and endocrine system, leading to behavioral impairments associated with mental health and neurodevelopmental disorders. Also, an early nutritional intervention (consumption of the control diet at weaning) was not sufficient to ameliorate many of the behavioral changes, such as increased anxiety, that were induced by maternal HFD consumption. Given the level of dietary fat consumption and maternal obesity in developed nations these findings have important implications for the mental health of future generations.

## Introduction

Developed nations have experienced a surge in the prevalence of both maternal obesity and pediatric neurodevelopmental disorders. In the United States, 64% of women of reproductive age are overweight, 35% are obese (1), and the majority of the population consumes excess dietary fat. The prevalence of obesity in pregnant women is particularly concerning, as the intrauterine and early postnatal environments are known to have a long-term impact on both the physiology and behavior of offspring. Early development is a sensitive period in which epigenetic changes can result in permanent alterations of behavioral and physiological processes. Given the prominent role that maternal nutrition and energy status play in regulating reproductive physiology, factors such as food availability, diet quality, and body weight are prime candidates for initiating epigenetic influences on offspring behavior and physiology. In epidemiologic studies, maternal obesity is associated with future risk of increased offspring Body Mass Index (BMI) (2), adiposity (3, 4), and metabolic disorders (3). Furthermore, maternal obesity and consumption of a high-fat diet (HFD) are associated with increased future risk of mental health and neurodevelopmental disorders (5, 6), such as attention-deficit hyperactivity disorder (ADHD) (5, 7, 8) and autism spectrum disorders (ASD) (9–11). Maternal obesity is also associated with childhood affective problems, such as increased risk of fear, sadness, and internalizing behavior (8, 12), and is correlated with low or high birth weight which are linked to anxiety and depression during adolescence (13). Both non-human primate (NHP) and rodent studies demonstrate that chronic maternal HFD consumption produces long-term alterations in offspring’s anxiety-related behaviors (6, 14).

It is challenging to study maternal diet in human participants due to difficulty in accurately monitoring food intake, and ethical issues related to manipulating the diet of pregnant women. Animal models have the advantage of investigator control over diet composition and elimination of many confounding genetic and environmental factors. Therefore, well-controlled animal studies are essential for exploring the specific effects of maternal diet and metabolic status on offspring behavior. NHP models are advantageous as they have complex social and mental health-related behaviors, similar developmental ontogeny of the brain and placental structure, and develop the full spectrum of metabolic disease consistent with humans. Using a NHP model of maternal HFD consumption, our group has shown that intrauterine overnutrition negatively impacts fetal development, resulting in increased activation of inflammatory cytokines in the placenta (15) and hypothalamus (16), decreased pancreatic α-cell plasticity (17), and abnormal development of the melanocortin system (16). We also documented changes in histone deacetylase activity in the liver of HFD offspring leading to decreased SIRT1 protein levels (18), suggesting NHP offspring are vulnerable to inflammation-induced epigenetic changes.

Our group further demonstrated perturbations of the NHP serotonergic system in the fetal brain and increased anxiety in infant female offspring from mothers consuming a HFD compared to infants exposed to a control diet (6). The purpose of the current study was to determine if these early alterations in behavior and brain development persist later in life. A second goal was to determine if an early intervention to a healthy diet at weaning would ameliorate maternal HFD-induced changes in behavior and brain development. In this study, we demonstrate that maternal and postweaning HFD consumption results in behavioral changes, including an increase in anxiety, that persist into the juvenile time period. These behavioral changes were associated with alterations in plasma and hair cortisol and impaired development of the central serotonin system, such as altered tryptophan hydroxylase-2 (TPH2) mRNA expression in the dorsal (DR) and median raphe (MnR) and reduced serotonin positive fibers in the medial prefrontal cortex (PFC). *Importantly, this is the first study to examine the long-term impact of HFD exposure during development on behaviors related to psychopathology in a NHP species*.

## Materials and Methods

### Animals

All animal procedures were in accordance with National Institutes of Health guidelines on the ethical use of animals and were approved by the Oregon National Primate Research Center (ONPRC) Institutional Animal Care and Use Committee. An in-depth characterization of the maternal (19) and juvenile (17, 20) phenotype has been previously reported.

#### Dietary Information

Macronutrient composition of the control diet (CTR) (Monkey Diet no. 5000; Purina Mills) and the HFD (TAD Primate Diet no. 5LOP, Test Diet, Purina Mills) are provided in Table 1. The diets has also been described comprehensively in previous publications (21). Monkeys on the HFD were also provided with calorically dense treats (35.7% of calories from fat, 56.2% of calories from carbohydrates, and 8.1% of calories from protein) once daily. The HFD represents a typical Western diet in respect to percent of calories provided by fat and saturated fat content.

**Table 1:** Macronutrient composition of experimental diets.

	Percent of diet		Percent of energy	
	CTR	High-fat diet (HFD)	CTR	HFD
**Protein**	**20.6**	**17.0**	**26.8**	**18.4**
**Fat**	**5.0**	**15.0**	**14.7**	**36.6**
Saturated	0.89	5.42		
Polyunsaturated	3.3	2.8		
*Linoleic*	*2.6*	*2.5*		
*Linolenic*	*0.34*	*0.10*		
*Arachidonic*	*0.0*	*0.06*		
*Omega-3*	*0.36*	*0.21*		
Monounsaturated	1.1	6.2		
**Carbohydrates**	**44.8**	**41.5**	**58.5**	**50.0**
Glucose	0.15	0.04		
Fructose	0.19	5.5		
Sucrose	2.8	8.8		
Lactose	0.0	4.6		
Starch	26	20.5		

*CTR = Monkey Diet no. 5000; Purina Mills. HFD = TAD Primate Diet no. 5LOP, Test Diet, Purina Mills. Macronutrient information provided from diet specification sheets*.

#### Adult Females

Adult female Japanese macaques (*Macaca fuscata*) were housed in groups of 4–12 individuals (male/female ratio of 1–2/3–10) in indoor/outdoor pens and were given *ad libitum* access to water and either the CTR or the HFD. Mothers in our study were aged 4.1–16.1 years and weighed 6.35–17.7 kg prepregnancy. Maternal body fat was determined using dual-energy X-ray absorptiometry prior to each pregnancy. Animals were sedated with Telazol (3–8 mg/kg; i.m., Fort Dodge Animal Health, Fort Dodge, IA, USA), supplemented with Ketamine HCl (5–10 mg/kg, i.m.; Ketaset, Fort Dodge Animal Health, Fort Dodge, IA, USA), and then positioned prone on the bed of a Hologic QDR Discovery scanner (Hologic, Bedford, MA, USA). Total body scans were done in the “Adult Whole Body” scan mode. Hologic QDR software version 12.6.1 was used to calculate body composition. Maternal body weight was assessed before pregnancy and during the third trimester of pregnancy. Percent weight gain during pregnancy was calculated by subtracting prepregnancy body weight from pregnancy body weight, dividing by prepregnancy body weight and multiplying by 100.

Demographic classification of maternal metabolic profiles is provided in Table 2. As expected, chronic consumption of a HFD produced elevations in percent body fat and body weight in our adult females. We noted an increase in fasting insulin and a normal fasting glucose in HFD dams. The high fasting insulin accompanied by normal fasting glucose indicates that our HFD dams are in the early stages of insulin resistance, but are not diabetic (22). Likewise, we observed an increase in insulin area under the curve (IAUC) and lower glucose area under the curve (GAUC) in HFD dams providing additional support that the HFD dams are becoming insulin resistant as they released more insulin to clear the glucose load administered during the glucose tolerance test (GTT). Several studies indicate that changes in insulin secretion occur prior to changes in blood glucose in patients that later develop type two diabetes (23, 24). It is also possible that the hyperinsulinemia is a response to the high circulating fatty acids due to HFD consumption, as insulin also regulates blood fatty acid levels (25).

**Table 2:** Demographic characteristics of CTR and high-fat diet (HFD) dams.

	CTR		HFD		
	Mean	SEM	Mean	SEM	*p*-Value
Age at birth (years)	9.70	0.33	9.23	0.26	0.297
Pregnancy number	3.77	0.26	3.83	0.20	0.544
Prepregnancy weight (kg)	10.0	0.26	10.9	0.30	0.013*
Gestational weight gain (percent)	6.72	1.36	4.86	1.61	0.404
Prepregnancy percent body fat	19.3	1.04	25.2	1.36	0.001*
Third trimester GAUC	7,375	136	6,613	176	0.002*
Third trimester IAUC	5,793	1,278	7,804	979	0.035*
Third trimester baseline glucose (mg/dl)	40.8	1.21	42.6	1.23	0.347
Third trimester baseline insulin (µU/ml)	26.5	7.53	40.5	4.63	0.00007*
Third trimester baseline leptin (ng/ml)	56.6	6.87	94.4	14.2	0.012*
Third trimester baseline glucagon (pg/ml)	85.6	5.93	88.8	5.23	0.519
*Sample size*	*64*		*71*		

*denotes difference between CTR and HFD dams, p < 0.05.

*GAUC, glucose area under the curve; IAUC, insulin area under the curve*.

*Maternal diet group differences were calculated from independent variables t-test for parametric measures and from a Mann–Whitney test for non-parametric measures*.

#### Juvenile Offspring

Offspring were born naturally from CTR or HFD mothers who had been consuming the diet for 1.2–8.5 years at time of parturition. The 135 offspring included in the study were born from 65 mothers, with no more than six offspring from the same mother. By 4 months of age infants began independent ingestion of the maternal diet and were consuming this diet as their primary food source by 6 months of age. Offspring were maintained with their mothers until the time of weaning at a mean of 7.99 months of age (SEM = 0.09). The offspring were then placed into group housing with 6–10 similarly aged juveniles and 1–2 adult females. Half of the offspring were maintained on their mothers’ diet and the other half switched diets, creating four diet groups (CTR/CTR, CTR/HFD, HFD/CTR, and HFD/HFD). The same animals were used for the majority of experimental measures; however, the actual sample size varied between measures. Only a subset of animals was euthanized at the 13-month time point for tissue collection, and as the data in this study were collected over 9 years some measures were added to the protocol in later years. The sample sizes for each group for the various measures are described in the figure legends.

### Behavioral Testing

At 11 months of age (average age 10.84 months, SEM = 0.025), juveniles were removed from their pens and placed in a cage located in an adjacent room between 0800 hours and 0830 hours. Individuals were then transported in a covered transfer box to the behavioral testing suite where they were placed in a standard primate cage. The juvenile was videotaped from an adjoining room through a one-way mirror for the duration of the test. The behavior tests were initiated between 0830 hours and 1100 hours.

#### Human Intruder Test

This test reliably evaluates individual differences in anxiety and stress response in NHPs (26, 27). The test began with a 10-min acclimation period, followed by a 2-min control period, and then a 2-min profile period, in which a human intruder (a woman unfamiliar to the monkey) entered the test room, stood 0.3 m from the cage, and presented her facial profile (a non-threatening stimulus) to the monkey. This was followed by another 2-min control period in which the stranger exited and the juvenile was alone. The human intruder then returned to the room for the 2-min stare period, stood in the same position 0.3 m from the cage, but made continuous direct eye contact (a threatening social stimulus) with the monkey. Another 2-min control period followed; the stranger reentered the room and made continuous direct eye contact while simultaneously offering a piece of apple (a desirable familiar food) to the monkey for the 2-min apple offer period.

#### Novel Objects Test

Anxiety-like behavior has been assessed in a variety of species, including NHPs, using novel object tests (28, 29). The test began after a 2-min control period following the human intruder test. For the first novel object, the human intruder entered, avoiding direct eye contact with the monkey, and placed a potentially threatening toy on the tray attached to the cage. A rubber ball with large eyes painted on it was used the first year the tests were done and a plastic toy bunny with large eyes for the following years. The toy was placed with the eyes facing the monkey. After 5 min, the human intruder reentered, removed the toy, and placed a pretzel (a novel food) on the tray attached to the cage. The monkey was left with the pretzel for 2 min, at which time the test concluded. The monkey was then hand caught to collect a blood sample for measurement of cortisol, before being placed in the transfer box and returned to their normal social housing.

#### Video Scoring

The videos of the behavior tests were scored using the Observer XT software Version 11 (Noldus Information Technology) *via* the continuous sampling method by two observers blind to maternal and postweaning diet. Behaviors such as locomotive state, exploration, vocalizations, anxious or abnormal behaviors, responses to stranger, and response to objects were scored. Actions which did not clearly fit a preestablished behavioral definition were coded as other and were submitted to a second blind observer for consideration. If agreement was not reached on the categorization of an individual’s behavior, the behavior was not included in video scoring. Behaviors were coded as one of two mutually exclusive categories: point events, behavioral events of no quantifiable duration, and state events, behavioral events of determined duration with a distinct beginning and ending.

#### Composite Variable Determination

Stress responses elicited in reaction to the behavioral tests vary widely between individuals; however, our subjects exhibited deviations from typical manifestations of anxiety, including the near-absence of displacement behaviors. This prompted the creation of more inclusive anxiety measures derived from video scoring results. In order to form cohesive anxiety composites, the frequency, distribution, and co-morbidity of functionally similar behaviors determined to be the result of stress were examined. Resulting behavioral composites are unweighted sums of percent duration and total number of component behaviors and are detailed in Table 3.

**Table 3:** Definition of behaviors used in the coding of the 11-month behavioral assessment.

Composite behaviors			Behavior	Definition	Occurrence in subjects (%)
			Locomotion	To move from one location to another, including shifting entire body (typically going from sitting to standing or *vice versa*) without taking a full step in any direction	100.0
			Jump^a^	To leap or jump as part of locomotion, with no limbs touching the cage	61.3
Active	Anxiety	Active anxiety	Stereotypy^b^	Abnormal pattern of movement repeated at least three consecutive times (examples include pacing, flipping, and twirling). If at least three repetitions occur, stereotypy begins with the start of the first repetition and ends when the pattern is broken	24.0
			Abnormal movement	Abnormal repetitive movement (flipping, rolling, jumping in place, pacing, rapid backing up, etc.) that cannot be considered stereotypy either due to brief breaks in the pattern or only two repetitions	34.7
			Other active anxiety^b^	Instances of atypical movement, commonly ritualized with continued recurrence throughout multiple periods of test. Often presented as single iterations of common stereotypic behavior (rock, jump/bounce, spin) or directed, forceful contact with side of cage, without being self-injurous	25.3
			Roll	Inversion of body into atypical position with persistent movement, often resulting in a roll onto cage floor	18.7
			Cage bite^b^	Forcefully and aggressively bite the cage in a non-exploratory manner	41.3
			Cage shake^a^^,^^b^	Grasp and shake the bars of the cage	14.7
			Escape	Forcefully attempting to push body through the cage, typically the feed slot or cage corner	22.7
			Abnormal vocalization^c^	Six or more vocalizations in immediate succession, beginning at first vocalization and ending when vocalization ceases even for a brief pause	4.0
			Teeth grind^b^	Clenching and grinding of teeth to produce an audible clicking/grinding sound	38.7
		Inactive anxiety	Freeze^d^	Suddenly stop all movement (often mid-stride) for three or more seconds. Typically in response to a threatening stimuli, such as the stranger. Ends with any change in body position, including minor head movement	89.3
Inactive			Shake^a^^,^^b^	Rapidly shake full body as if to remove water particles (resembles a wet dog shake)	16.0
			Scratch^a^^,^^b^	Use fingers or toes to scratch own body	6.7
			Yawn^a^^,^^b^	Opening the mouth widely. Can be differentiated from open mouth threats by a lack of eye contact with another subject	0.0
			Fear grimace^a^^,^^b^	Draw back lips to display clenched or slightly parted teeth	1.3
			Abnormal posture^b^	Maintain an atypical body position for three or more seconds. Most typically seen as hyperextension of the head while sitting, standing, or hanging	28.0
			Lay down	To be laying on one’s side or back for two or more seconds	10.7
			Sleep	Stationary with eyes fully closed for three or more seconds, seemingly asleep	2.7
			Other abnormal^b^	Abnormal behavior that is not otherwise listed. Examples include self biting, self hitting, hair pulling, eye poking, and floating limb	1.3
			Self comfort^b^	Group of behaviors including mouthing/sucking own digits or wrapping arms around torso and embracing/clasping self. Usually occurs with cessation of other behaviors and marked introversion	0.0
			Self groom^b^	Pick through or lick own fur/skin or biting own nails. May include placing debris from coat into mouth	28.0
			Crouch^b^	Lowering entire ventral surface to the floor of the cage for three or more seconds, typically in response to a stimulus	33.3
			Stationary	To be still and not changing location for two or more seconds, and not engaging in any other primary behavior	100.0
			Vocalization^a^^,^^b^	Bark, chirp, coo, grunt, and shriek	32.0
			Explore^b^	Purposefully interacting with cage using mouth or hands	98.7
			Interact with objects	Intentional physical contact with an object, using hands, feet, or mouth, including eating	96.0
			Vigilant	Keeping the stranger or toy within direct or peripheral vision	100.0

*^a^Denotes point events (behavioral events of no quantifiable duration) not included in percent duration analyses*.

*^b^Winnicker et al. (30)*.

*^c^Gorman (31)*.

*^d^Coleman and Pierre (32)*.

*Freeze was excluded from Inactive composite because characterization of type of inactivity was always simultaneously coded (stationary, hang, crouch)*.

### Activity Measurements

Physical activity levels were measured using accelerometers (Actical, MiniMitter, Bend, OR, USA) mounted on plastic collars (Primate Products, Miami, FL, USA) worn continuously by juveniles after weaning. This method for measuring physical activity in NHPs is well established and previously reported in detail (33). Activity levels were collected during the behavior test and in normal social housing the day prior to and following behavioral testing. This allowed examination of the effect of behavioral testing on activity during the testing interval and longer term alteration in activity.

### Glucose Tolerance Tests

An intravenous GTT was performed on the dam at the third trimester of each offspring’s gestational period (average 47.3 days before parturition, SEM = 0.72), and on the offspring at 13 months (average age 12.98 months, SEM = 0.069). Animals were fasted overnight prior to the GTT. The morning of the procedure the animal was removed from their pen and placed in a cage in an adjacent room between 0800 hours and 0830 hours, receiving no food once removed from the group. The animal was then sedated with Telazol (5 mg/kg i.m.), and after 10 min of deep sedation, a baseline sample of 3–5 ml of blood was collected from a catheter placed in the right saphenous vein. Of this sample, 0.5 ml was used to saturate a glucose test strip placed in a OneTouch Ultra2 Blood Glucose Monitor (LifeScan, Milpitas, CA, USA) to record the baseline glucose level. The remainder of the blood was kept in heparinized tubes on ice for insulin, glucagon, and leptin measurements. A glucose bolus (50% dextrose solution) at a dose of 0.6 g/kg was administered intravenously via the right saphenous catheter. Further glucose measures were recorded from 0.5 ml blood samples collected from the left saphenous vein at 1, 3, 5, 10, 20, 40, and 60 min after infusion. The remainder of the blood was kept in heparinized tubes on ice for insulin measurements. After the GTT, samples were centrifuged, and plasma was stored at −80°C until assayed. All glucose and insulin measures from baseline until 60 min post-infusion were then used to calculate the area under curve (GAUC and IAUC, respectively) from 0 using GraphPad Prism Version 6 software (GraphPad Software, Inc., La Jolla, CA, USA).

### Blood Sample Collection

Juvenile blood samples were collected from the femoral or saphenous vein. Blood was collected into a heparin tube, which was placed on ice and centrifuged at 1,125 × *g* at 4°C for 20 min. Plasma was removed and stored at −80°C until assay.

#### Preweaning Samples

At 3 months of age (average age 90.27 days, SEM = 0.223) dam and infant pairs were removed from their pens and placed in a cage in an adjacent room between 0800 hours and 0830 hours. The infant was then immediately separated from the dam for a 30-min period. Following this period, the infant was sedated with Ketamine (5–10 mg/kg i.m.) and a 1–2 ml blood sample was collected from the femoral or saphenous vein.

At 4 months of age (average age 129.48 days, SEM = 0.398) dam and infant pairs were removed from their pens and placed in a cage in an adjacent room between 0800 hours and 0830 hours. The infant was separated from the dam and transferred in a covered transport box to the behavioral testing suite where they were placed in a standard primate cage. The infant received a behavioral test consisting of a human intruder test and novel objects test, which has been previously detailed (6). Immediately following the behavioral test the infant was hand caught from the cage and restrained while a 2-ml blood sample was collected from the femoral vein. All samples were collected within 5 min following the conclusion of the behavioral test.

Prior to weaning (average age 180.22 days, SEM = 0.512) dam and infant pairs were removed from their pens and placed in a cage in an adjacent room between 0800 hours and 0830 hours. The infant was separated from the dam until 1200 hours. The infant was then sedated with Telazol (3 mg/kg i.m.), and 2 ml of blood was collected from the saphenous vein.

#### Postweaning Samples

Immediately following the aforementioned 11-month behavioral test, the juvenile was hand caught from the cage in the behavioral testing suite. The juvenile was then restrained while a 2 ml blood sample was collected from the femoral vein. All samples were collected within 5 min following the conclusion of the behavioral test.

### Hair Sample Collection

Hair was collected prior to weaning (average age 7.06 months, SEM = 0.130) and at 13 months (average age 13.10 months, SEM = 0.089). The animals were sedated with either Ketamine (5–10 mg/kg i.m.) or Telazol (5 mg/kg i.m.) and a hair sample was collected from the right subscapular region. The hair sample was placed in an aluminum foil packet and frozen at −80°C until the time of assay.

### Tissue Collection and Processing

Offspring were necropsied at 13 months of age, and brain tissue was collected as previously described (6, 34, 35). Euthanasia was performed by ONPRC Necropsy staff and adhered to AVMA Guidelines on Euthanasia in Animals and ONPRC standard operating procedures and guidelines. Animals were sedated with Ketamine (15–25 mg/kg i.m.) and transported to the necropsy room in a covered transport box. The animals were deeply anesthetized with a surgical dose of sodium pentobarbital (25–35 mg/kg i.v.). Anesthetic depth was monitored by assessing the loss of palpebral, corneal, pain, and pharyngeal reflex. After adequate plane of anesthesia was reached, the abdomen was incised and terminal blood samples were collected from the aorta or caudal vena cava. The aorta was then severed and the animal exsanguinated. Perfusion of the brain occured via the carotid artery by flushing with 0.9% heparinized saline (0.5–1 l) followed by 4% paraformaldehyde (PF, approximately 1–2 l) buffered with sodium phosphate (NaPO4, pH 7.4) until fixed. The brain was then partitioned into specific areas and placed in 4% PF for 24 h at 4°C, transferred to 10% glycerol buffered with NaPO4 for 24 h, and finally transferred to 20% glycerol solution for 72 h. Tissue blocks were frozen in −50°C 2-methylbutane and then stored in −80°C until sectioning.

### Plasma Assays

The ONPRC Endocrine Technologies Support Core (ETSC) at the ONPRC performed assays for cortisol (17α-hydroxycorticosterone) and insulin using a chemiluminescence-based automatic clinical platform (Roche cobas e411, Roche Diagnostics, Indianapolis, IN, USA) validated for NHP serum and plasma (36). Company-provided calibrators and quality control samples were analyzed before each use. The intra- and inter-assay variation of the assay for cortisol was less than 7% and the assay range was 0.36–63.40 ng/ml. The intra- and inter-assay variation of the assay for insulin was less than 7% and the assay range was 0.2–1,000 μIU/ml. Fasting glucagon was assayed by radioimmunoassay (RIA) (Catalog no. GL-32K; Millipore). The intra-assay variations were less than 8% and the assay range was 20–4,000 pg/ml. As all samples were analyzed in a single assay for each target, no specific inter-assay variations for this study were calculated. Leptin levels were measured using an RIA kit directed against human leptin (Catalog no. HL-81K; Millipore). The intra-assay variations were less than 17% and assay range was 0.78–100 ng/ml. Overall inter-assay variations for the leptin and glucagon RIAs in the ETSC are less than 20%.

### Hair Assays

Cortisol was measured in a hair sample to measure chronic stress. Hair cortisol reflects the mean cortisol over the past several weeks to months (37). The ETSC at ONPRC analyzed cortisol in hair samples using a modification of an existing protocol (37). Hair was washed with isopropanol (5 ml), filtered with P8 filter paper (Fisher Brand cat. No.: 09-795D), and minced manually with a specially designed multi-blade cutter with blade distance at 2 mm. Next, the cortisol was extracted by gentle shaking in methanol (50 mg/ml) for approximately 22 h. Hair and methanol were then separated by centrifugation and the supernatant was collected and dried under a forced air stream at 45–50°C. Finally, the dried contents were reconstituted in assay buffer and cortisol levels determined by ELISA (Salimetrics, State College, PA, USA). Recoveries were determined at the same time as sample analysis and used to adjust final sample cortisol values. Intra-assay variation was less than 10% and inter-assay variation was less than 15% (*n* = 5). The assay range was 0.33–30.00 ng/ml.

### *In Situ* Hybridization

Tissues were processed for *in situ* hybridization, as previously detailed (6, 34). Midbrain blocks from offspring necropsied at 13 months were sectioned using a freezing microtome at 25 µm, collected in 1:24 series, and stored at −20°C in ethylene glycol cryoprotectant. Sections in 1:24 series were slide mounted in potassium phosphate-buffered saline (KPBS) (pH 7.4), and dried overnight by vacuum-dessication. cRNA probes were transcribed in the presence of 100% P33-labeled UTP (PerkinElmer, Waltham, MA, USA) from cDNA clones of tryptophan hydroxylase-2 (TPH2; 300 bp), serotonin transporter (SERT; 253 bp), and serotonin 1A receptor subtype (5-HT1AR; 431 bp). Probe-labeled sections were exposed to film for 2d (SERT), 2d (TPH2), or 5d (5-HT1aR) for visualization. A CoolSnap HQ camera and MetaMorph software were used to capture autoradiographic images, which were analyzed by integrated morphometry analysis to measure perecent area and density. Total density was determined by multiplying total area by optical density of each level of the midbrain (rostral ≈ bregma—17.78 ± 1 mm, medial ≈ bregma—19.75 ± 1 mm, and caudal ≈ bregma—23.40 ± 1 mm). Three to four matched sections were analyzed for each midbrain level in each monkey.

### Immunohistochemistry for Serotonin

Coronal sections (25 µm) of the right PFC were collected in 1:24 series using a freezing microtome, as previously described (34). Briefly, sections were washed in KPBS and then blocked in 2% donkey serum in 0.4% Triton-X into KPBS for 30 min. Rabbit anti-5-HT (Lot #082M4831, #S5545; 1:5,000; Sigma-Aldrich) antibody was diluted in 2% donkey serum in 0.4% triton X into KPBS and applied to tissue sections which were incubated at room temperature for 1 h and then at 4°C for 48 h. Tissues were then washed in KPBS, and the secondary antibody (Donkey-Anti-Rabbit Alexa Fluor 488; Lot #1531671 Life Technologies Corporation, Carlsbad, CA, USA) was applied for 1 h at room temperature at a dilution of 1:1,000. Sections were washed again in KPBS, and then counterstained using DAPI (Molecular probes cat# D-1306) diluted in KPBS and Triton-X for approximately 30 s. Sections were washed once more and then mounted on gelatin subbed slides and coverslipped with Slowfade Mountant (Life Technologies Corporation, Carlsbad, CA, USA) and stored at 4°C.

**Figure 1:**
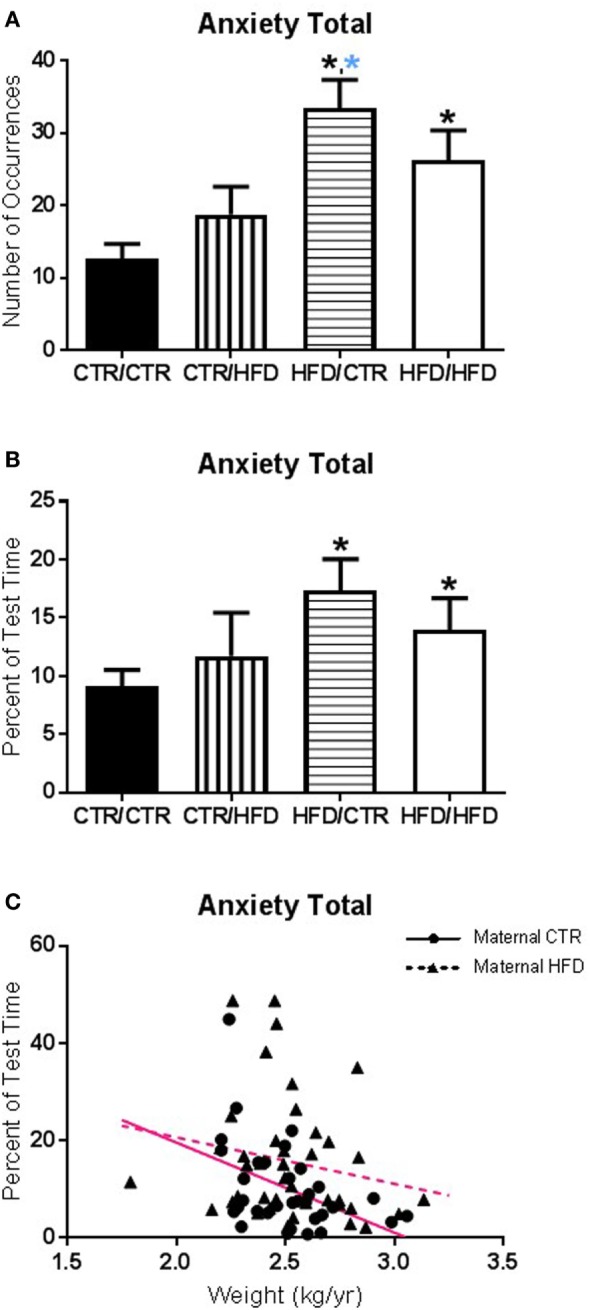
**(A)** The number of anxiety behaviors during the 11-month behavioral assessment increased with exposure to maternal high-fat diet (HFD) during perinatal development (*F*_1,67_ = 12.098, *p* = 0.001) and was affected by an interaction between maternal and postweaning diet (*F*_1,67_ = 4.662, *p* = 0.034). The HFD/CTR group performed more anxiety behaviors than CTR/CTR animals (*F*_1,67_ = 21.276, *p* = 0.000018). **(B)** Controlling for offspring weight, the amount of time spent performing anxiety behaviors increased with maternal HFD exposure (*F*_1,65_ = 4.498, *p* = 0.038). **(C)** The amount of time engaging in anxiety behaviors was associated with offspring body weight; offspring with lower body weight spent more time engaged in anxiety behaviors (*F*_1,65_ = 5.819, *p* = 0.019). Data shown as mean ± SEM. A black * denotes maternal diet effect, *p* < 0.05. A blue * denotes a maternal diet effect when controlling for postweaning diet. Magenta lines denote significant overall covariance, *p* < 0.05. Sample sizes are as follows: CTR/CTR *n* = 21 (*n* = 10 males; *n* = 11 females), CTR/HFD *n* = 12 (*n* = 8 males; *n* = 4 females), HFD/CTR *n* = 23 (*n* = 12 males; *n* = 11 females), and HFD/HFD *n* = 18 (*n* = 8 males; *n* = 10 females).

**Figure 2:**
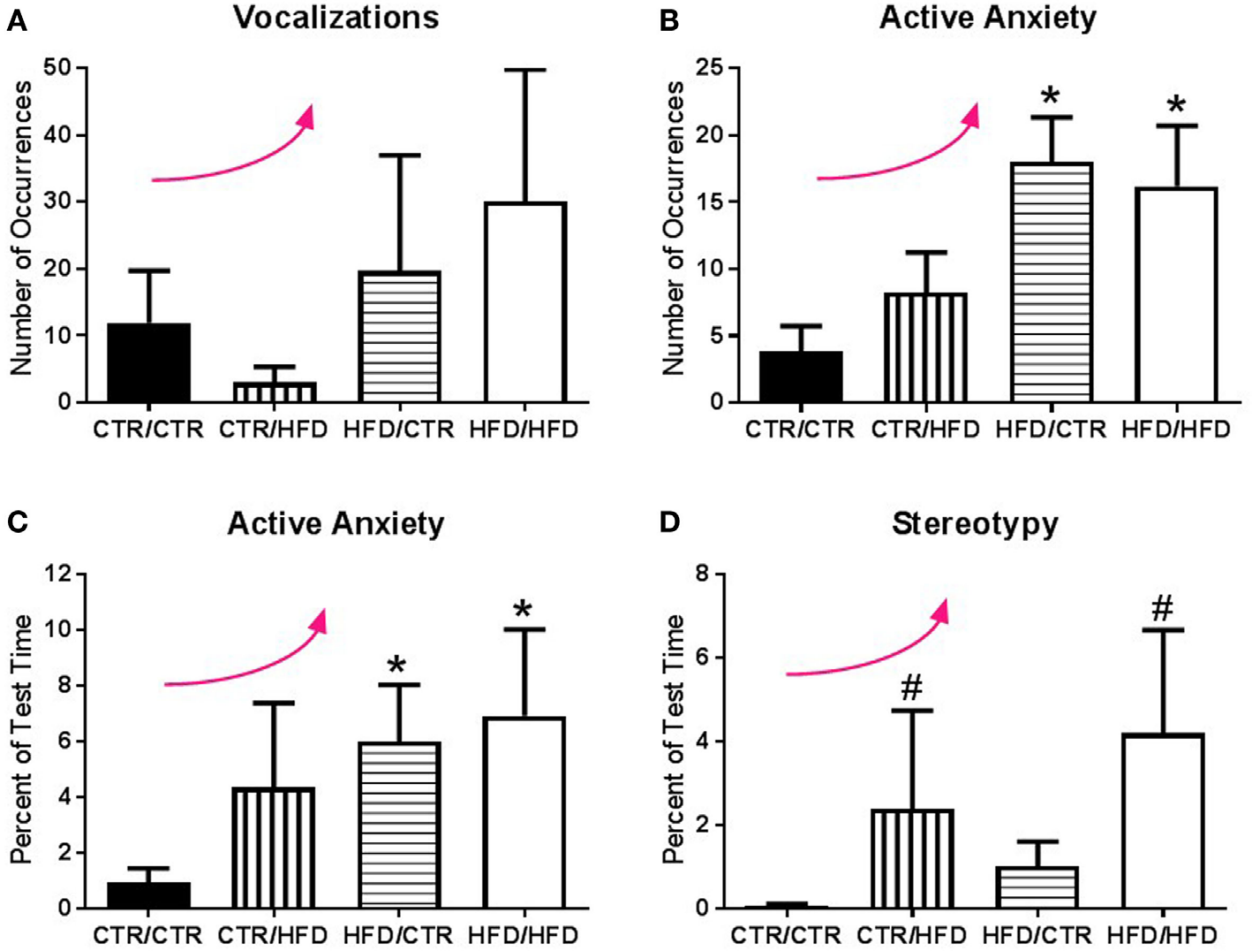
**(A)** Jonckheere’s tested revealed the number of vocalizations exhibited during the 11-month behavior test increased with high-fat diet (HFD) exposure (*p* = 0.033). **(B)** Exposure to a maternal HFD increased the number of active anxiety behaviors (*p* = 0.0006), in both male (*p* = 0.042) and female (*p* = 0.015) offspring. Jonckheere’s test detected an interaction between maternal diet and postweaning diet, with occurrences of active anxiety increasing with exposure to the HFD (*p* = 0.0004). **(C)** The percent of time exhibiting active anxiety behaviors likewise increased with a maternal HFD (*p* = 0.003). Postweaning HFD also impacted the percent of time exhibiting active anxiety with active anxiety increasing with increased exposure to the HFD (*p* = 0.001). **(D)** Animals exposed to a postweaning HFD displayed increased levels of stereotypy (*p* = 0.027). There was also a significant trend with exposure to HFD increasing the percent of time exhibiting stereotypy (*p* = 0.002). Data shown as mean ± SEM. * denotes maternal diet effect, ^#^ denotes a postweaning diet effect, and a curved arrow indicates a significant trend detected by a Jonckheere’s test, *p* < 0.05. Sample sizes are as follows: CTR/CTR *n* = 21 (*n* = 10 males; *n* = 11 females), CTR/HFD *n* = 12 (*n* = 8 males; *n* = 4 females), HFD/CTR *n* = 24 (*n* = 12 males; *n* = 12 females), and HFD/HFD *n* = 18 (*n* = 8 males; *n* = 10 females).

**Figure 3:**
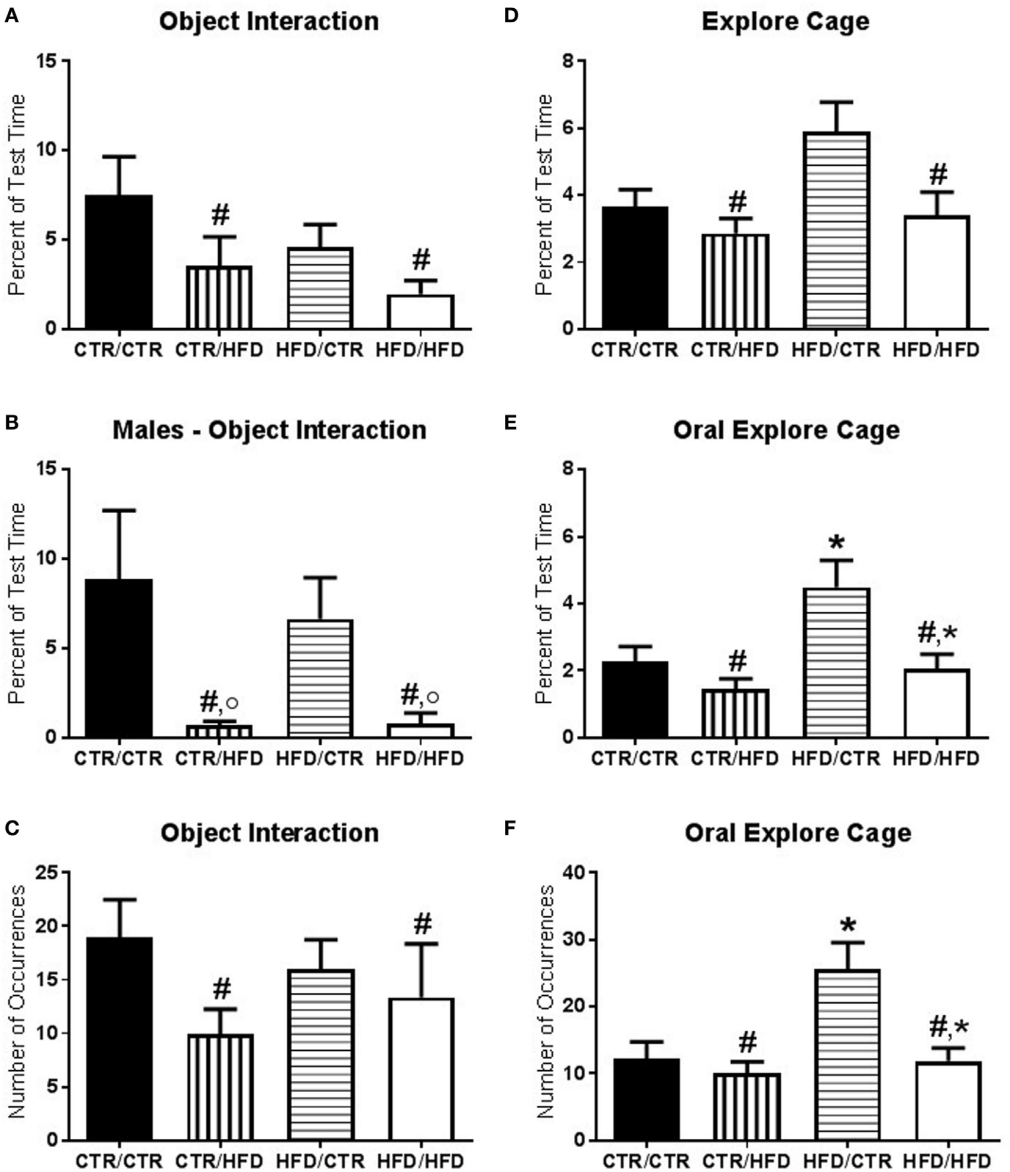
**(A)** The amount of time interacting with novel objects introduced during the behavior test decreased with postweaning high-fat diet (HFD) exposure (*F*_1,67_ = 4.874, *p* = 0.031). **(B)** There was an interaction between postweaning diet and gender (*F*_1,67_ = 13.613, *p* = 0.000425) with postweaning HFD males interacting less with the novel objects compared to CTR males (*F*_1,67_ = 19.213, *p* = 0.000042) and compared to HFD females (*F*_1,67_ = 14.862, *p* = 0.000262). **(C)** Postweaning HFD exposure decreased the number of interactions with novel objects (*F*_1,66_ = 5.340, *p* = 0.024). **(D)** Total time spent exploring the cage was reduced in postweaning HFD offspring (*F*_1,67_ = 6.639, *p* = 0.012). **(E)** Percent duration of oral exploration of the cage increased with maternal HFD exposure (*F*_1,67_ = 5.300, *p* = 0.024) and decreased with postweaning HFD exposure (*F*_1,67_ = 6.915, *p* = 0.011). **(F)** Total number of oral explore behaviors likewise increased with maternal HFD exposure (*F*_1,67_ = 4.350, *p* = 0.041) and decreased with postweaning HFD exposure (*F*_1,67_ = 4.415, *p* = 0.039). Data shown as mean ± SEM. * denotes maternal diet effect, ^#^ denotes a postweaning diet effect, and ° denotes a gender difference. *p* < 0.05. Sample sizes are as follows: CTR/CTR *n* = 21 (*n* = 10 males; *n* = 11 females), CTR/HFD *n* = 12 (*n* = 8 males; *n* = 4 females), HFD/CTR *n* = 24 (*n* = 12 males; *n* = 12 females), and HFD/HFD *n* = 18 (*n* = 8 males; *n* = 10 females).

**Figure 4:**
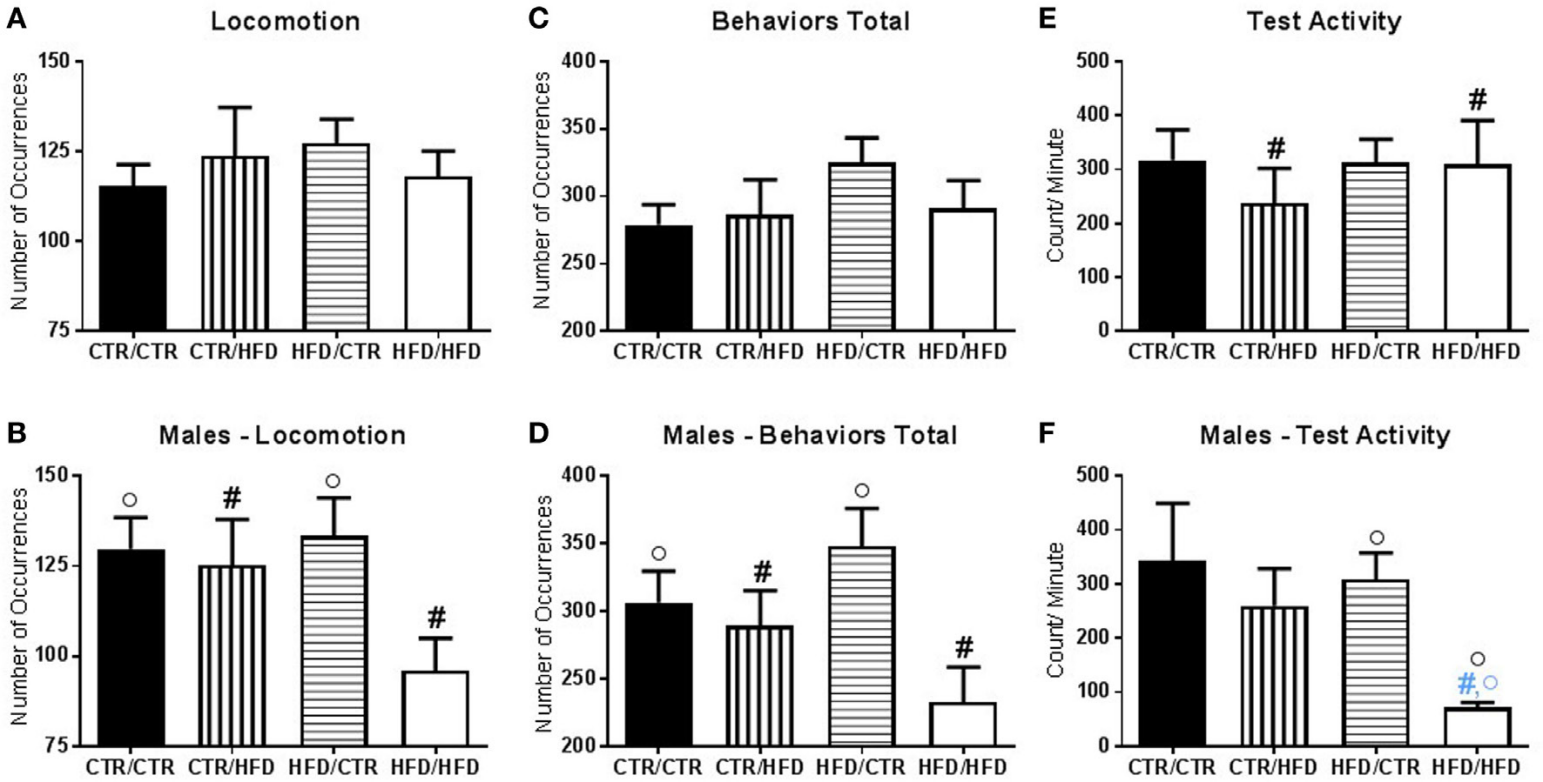
**(A)** The number of locomotive behaviors was affected by an interaction between postweaning diet and gender (*F*_1,67_ = 6.047, *p* = 0.017). **(B)** Postweaning high-fat diet (HFD) males exhibited fewer locomotive behavior compared to CTR males (*F*_1,67_ = 4.135, *p* = 0.046). Postweaning CTR male offspring displayed higher locomotive behaviors than females (*F*_1,67_ = 4.585, *p* = 0.036). **(C)** The total number of behaviors performed during the test was affected by an interaction between postweaning diet and gender (*F*_1,67_ = 6.451, *p* = 0.013). **(D)** In males, postweaning HFD consumption decreased the number of behaviors exhibited during the behavior test (*F*_1,67_ = 6.588, *p* = 0.013), and within postweaning diet CTR male offspring performed more behaviors than females (*F*_1,67_ = 4.383, *p* = 0.040). **(E)** Activity levels during the behavior test decreased with postweaning HFD exposure (*F*_1,37_ = 5.327, *p* = 0.027). **(F)** There was an interaction with gender and maternal diet (*F*_1,37_ = 5.252, *p* = 0.028), which showed that maternal HFD decreased activity in male offspring as compared to females (*F*_1,37_ = 4.406, *p* = 0.043). There was also an interaction with gender, maternal, and postweaning diet (*F*_1,37_ = 5.846, *p* = 0.021). HFD/HFD males displayed reduced activity compared to females (*F*_1,37_ = 7.447, *p* = 0.010) and HFD/CTR males (*F*_1,37_ = 6.548, *p* = 0.015), but not from CTR/HFD males (*F*_1,37_ = 4.042, *p* = 0.052). Data shown as mean ± SEM. A black ^#^ denotes a postweaning diet effect, and a black ° denotes a gender difference, *p* < 0.05. A blue ^#^ denotes a postweaning diet effect when controlling for maternal diet and gender and a blue ° denotes a gender effect when controlling for maternal and postweaning diet, *p* < 0.05. Sample sizes for the behavioral experiments are as follows: CTR/CTR *n* = 21 (*n* = 10 males; *n* = 11 females), CTR/HFD *n* = 12 (*n* = 8 males; *n* = 4 females), HFD/CTR *n* = 24 (*n* = 12 males; *n* = 12 females), and HFD/HFD *n* = 18 (*n* = 8 males; *n* = 10 females). Sample sizes for activity data are as follow: CTR/CTR *n* = 15 (*n* = 6 males; *n* = 9 females), CTR/HFD *n* = 8 (*n* = 7 males; *n* = 1 female), HFD/CTR *n* = 14 (*n* = 8 males; *n* = 6 females), and HFD/HFD *n* = 8 (*n* = 2 males; *n* = 6 females).

**Figure 5:**
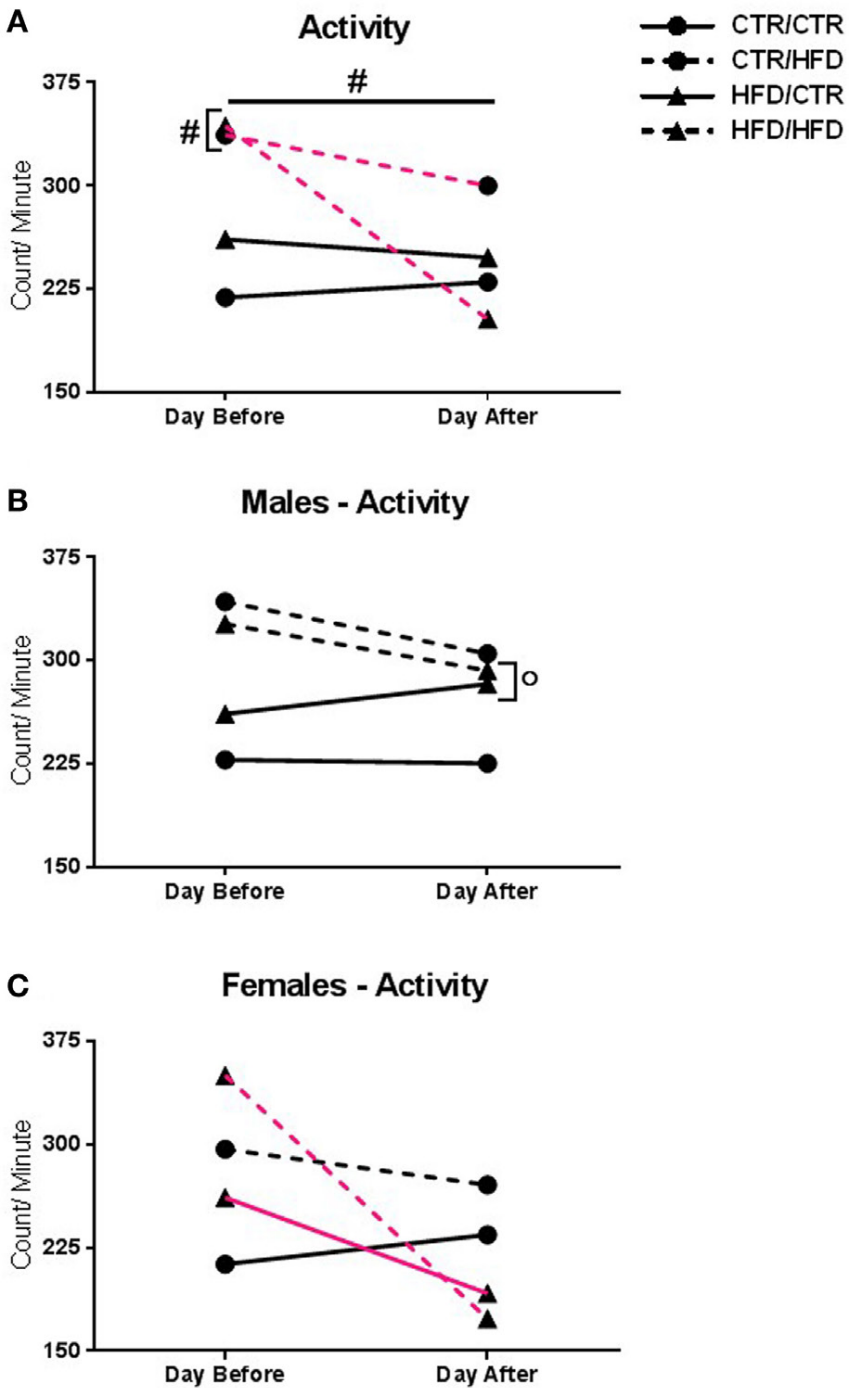
**(A)** Activity level was reduced the day after the behavior test in all offspring (*F*_1,31_ = 5.879, *p* = 0.021). Postweaning high-fat diet (HFD) increased activity (*F*_1,31_ = 8.701, *p* = 0.006). There was an interaction with day and postweaning diet (*F*_1,31_ = 4.868, *p* = 0.035), with postweaning HFD increasing activity the day before the test (*F*_1,31_ = 15.007, *p* = 0.001). Compared to their pre-test levels, postweaning HFD-exposed animals decreased activity after the test (*F*_1,31_ = 7.327, *p* = 0.011). **(B)** There was an interaction with day, gender, and maternal diet (*F*_1,31_ = 4.749, *p* = 0.037). After the test, maternal HFD males showed increased activity compared to females (*F*_1,31_ = 9.749, *p* = 0.004). **(C)** The interaction also showed that maternal HFD females had decreased activity compared to pre-test levels (*F*_1,31_ = 21.016, *p* = 0.00007), but not from controls (*F*_1,31_ = 2.957, *p* = 0.096). Data shown as mean ± SEM. ^#^ denotes a postweaning diet effect and ^°^ denotes a gender difference, *p* < 0.05. Magenta lines indicate significant difference of indicated diet group between days, *p* < 0.05. Symbols adjacent to data points indicate significant difference on that day only, *p* < 0.05. Sample sizes are as follows: Day before: CTR/CTR *n* = 15 (*n* = 6 males; *n* = 9 females), CTR/HFD *n* = 8 (*n* = 7 males; *n* = 1 females), HFD/CTR *n* = 13 (*n* = 8 males; *n* = 5 females), and HFD/HFD *n* = 7 (*n* = 2 males; *n* = 5 females). Day after: CTR/CTR *n* = 13 (*n* = 6 males; *n* = 7 females), CTR/HFD *n* = 7 (*n* = 6 males; *n* = 1 females), HFD/CTR *n* = 13 (*n* = 8 males; *n* = 5 females), and HFD/HFD *n* = 8 (*n* = 2 males; *n* = 6 females).

**Figure 6:**
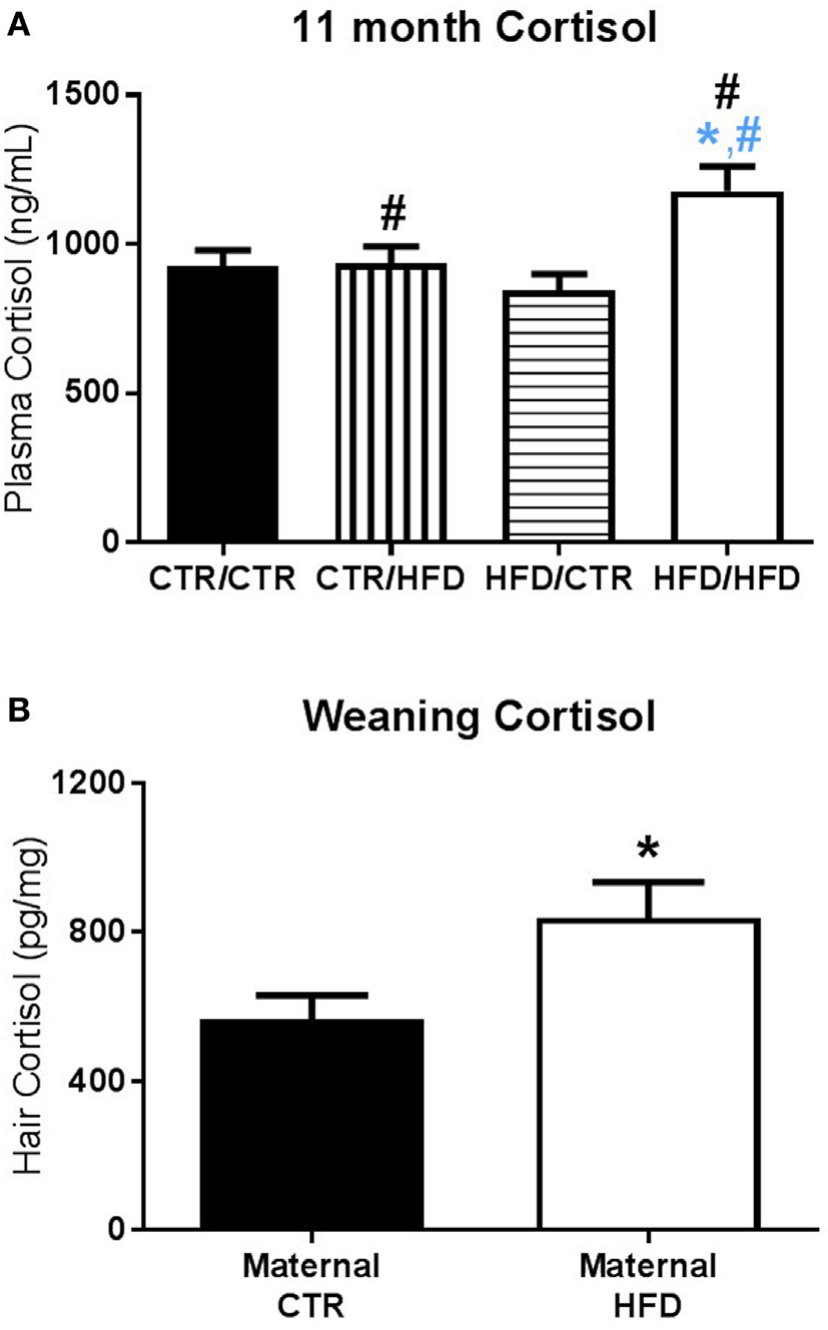
**(A)** Plasma cortisol measured after the 11-month behavior test increased with postweaning high-fat diet (HFD) exposure (*F*_1,83_ = 6.729, *p* = 0.011). Post-test cortisol was also affected by an interaction between maternal and postweaning diet (*F*_1,83_ = 5.772, *p* = 0.019), with the HFD/HFD group increased from HFD/CTR (*F*_1,83_ = 12.966, *p* = 0.001) and from CTR/HFD (*F*_1,83_ = 5.489, *p* = 0.022). Sample sizes for 11-month plasma cortisol are as follows: CTR/CTR *n* = 27 (*n* = 13 males; *n* = 14 females), CTR/HFD *n* = 18 (*n* = 11 males; *n* = 7 females), HFD/CTR *n* = 25 (*n* = 12 males; *n* = 13 females), and HFD/HFD *n* = 21 (*n* = 9 males; *n* = 12 females). **(B)** At weaning, offspring exposed to a maternal HFD displayed higher hair cortisol than controls (*F*_1,46_ = 4.406, *p* = 0.041). Sample sizes for weaning hair cortisol are as follows: maternal CTR *n* = 20 (*n* = 11 males; *n* = 9 females), maternal HFD *n* = 17 (*n* = 10 males; *n* = 7 females). Data shown as mean ± SEM. A black * denotes maternal diet effect and a black ^#^ denotes a postweaning diet effect, *p* < 0.05. A blue * denotes maternal diet effect when controlling for postweaning diet and a blue ^#^ denotes a postweaning diet effect when controlling for maternal diet, *p* < 0.05.

Serotonin immunofluorescent images were captured using Confocal laser microscopy on a Leica SP5 AOBS Confocal microscope (Leica Microsystems, Inc., Bannockburn, IL, USA) as previously described (38). We imaged six fields of view per section throughout area 10 of the right PFC, two fields of view each of the dorsal, medial, and ventral regions as defined by the Paxinos Stereotaxic Atlas (39, Figures 1–6) in anatomically matched sections for each animal. The observer was blind to maternal and postweaning diet when imaging slides. Images were taken at a format of 1,024 × 1,024, zoom factor 1, 400 Hz, in 2 µm increments along the *z*-axis of the tissue using a 10× (NA 0.40) objective. The 405-nm line of a blue diode laser and a 488-nm argon laser were used sequentially to avoid bleed-through of individual fluorophores into the nearby detection channels. ImageJ software (Wayne Rasband, National Institute of Health, Bethesda, MD, USA) was used to measure the total fluorescent intensity, the percent area and the integrated density of 5-HT by an individual blind to maternal and postweaning diet. DAPI staining was used to identify the layers and three measurements using an oval region of interest were taken of each layer (1–6) for each animal. These three regions were averaged for each layer of each image and were used to calculate the overall average fluorescent intensity, percentage area and integrated density of each layer for each animal.

### Statistical Analysis

Statistical tests were run using SPSS Version 22 (SPSS Inc., Chicago, IL, USA). For all variables, Kolmogorov–Smirnov tests of normality were run for the three pre-determined factors (maternal diet, postweaning diet, and gender) with *p* < 0.05 indicating a significant deviation from normality. If data were non-parametric a square root or log10 transformation was applied to obtain normally distributed data. Remaining non-parametric measures were rank transformed to achieve normality, with mean rank assigned to ties. Data are presented as mean ± SEM. Alpha values of *p* < 0.05 were considered statistically significant. All graphs were made with GraphPad Prism Version 6 software (GraphPad Software, Inc., La Jolla, CA, USA).

#### Parametric Analysis

Physical activity, cortisol, TPH2, SERT, and 5-HT1A mRNA expression, 5-HT immunohistochemistry, and select behavioral measures were determined to be parametric and tested for homogeneity of variance. The effect of juvenile metabolic state on observed outcomes was examined, using GAUC and body weight taken at the 13-month GTT as metabolic parameters. Pearson correlations were run, with *p* < 0.05 identifying potential covariates. Direct eye contact (*r* = 0.239, *p* = 0.048) and vigilance (*r* = −0.242, *p* = 0.046) during the 11-month behavior test, as well as MnR TPH2 percent area (*r* = 0.447, *p* = 0.007) and density (*r* = 0.451, *p* = 0.007) correlated with GAUC. Percent duration of anxiety behaviors during the 11-month behavior test correlated with weight (*r* = −0.294, *p* = 0.011). For these outcomes, the associated metabolic variable was used in three-factor univariate ANCOVAs. Change in activity was tested with a three-factor repeated measures ANOVA. Test activity, postweaning plasma and hair cortisol, dorsal raphe (DR) TPH2, SERT, and 5-HT1A mRNA expression, 5-HT immunohistochemistry measures, and remaining parametric behaviors were analyzed with a three-factor univariate ANOVA. Preweaning cortisol measures were analyzed with a two-factor univariate ANOVA. All pairwise analyses following ANOVA or ANCOVAs utilized Bonferroni corrections.

#### Non-Parametric Analysis

Measures which did not achieve normality by any transformation attempt underwent a series of tests to investigate the same three factors explored in parametric analysis. To examine juvenile metabolic effects Kendall’s correlations were run for GAUC and weight. Only latency to contact apple in the 11-month test correlated with weight (*r* = −0.232, *p* = 0.010), with no measures associated with GAUC. The relationship between metabolic parameters and observed outcomes, independent of variation due to maternal diet, postweaning diet, and gender was examined using Kendall’s partial correlations, with *p* < 0.05 indicating unique variance. No measures produced significant partial correlation results so group differences were examined independent of metabolic parameters. Variables were first tested using Mann–Whitney *U* tests to examine maternal diet, postweaning diet, and gender. Kruskal–Wallis tests were then performed on two sets of four independent groups, classified by an individual’s gender and maternal diet and their gender and postweaning diet, in order to examine the relationship between diet and gender. Last, the Jonckheere–Terpstra test was performed on diet groups, ordered with increasing exposure to the HFD, assessing the effect of HFD exposure across maternal and postweaning diets. Jonckheere–Terpstra tests for ordered pattern of medians across independent groups with meaningful order, such as our diet groups. Both Mann–Whitney and Kruskal–Wallis two-tailed *p*-values, as well as all follow-up pairwise examinations, were adjusted for number of comparisons. Jonckheere–Terpstra one-tailed *p*-values remain unadjusted.

## Results

### Impact of Exposure to a HFD during Development on Juvenile Offspring Behavior

#### Maternal and Postweaning HFD Consumption Increased Anxiety

The number of anxiety behaviors (Table 3) during the 11-month behavioral assessment increased with exposure to maternal HFD during perinatal development (*F*_1,67_ = 12.098, *p* = 0.001; Figure 1A), and was affected by an interaction between maternal and postweaning diet (*F*_1,67_ = 4.662, *p* = 0.034). Follow-up analysis showed the HFD/CTR group performed more anxiety behaviors than CTR/CTR animals (*F*_1,67_ = 21.276, *p* = 0.000018). ANCOVA results controlling for juvenile weight revealed that the amount of time spent performing anxiety behaviors also increased with maternal HFD exposure (*F*_1,65_ = 4.498, *p* = 0.038, Figure 1B). In addition, the amount of time engaging in anxiety behaviors was found to be associated with offspring body weight, such that offspring with lower body weight spent more time engaged in anxiety behaviors (*F*_1,65_ = 5.819, *p* = 0.019, Figure 1C). The number of vocalizations, an established measure of anxiety (32, 40), similarly increased with HFD exposure (*p* = 0.033, Figure 2A) according to Jonckheere’s test. Offspring exposed to maternal HFD displayed increased number of active anxiety behaviors (*p* = 0.0006, Figure 2B), which was maintained in males (*p* = 0.042) and females (*p* = 0.015). Jonckheere’s test detected an interaction between maternal and postweaning diet, with occurrences of active anxiety increasing with HFD exposure (*p* = 0.0004). Time exhibiting active anxiety likewise increased with maternal HFD (*p* = 0.003, Figure 2C). Moreover, step-down analysis revealed that any exposure to the HFD increased active anxiety (*p* < 0.05) compared to no exposure. Females exposed to maternal HFD exhibited more active anxiety than controls (*p* = 0.042). Stereotypy increased with postweaning HFD consumption (*p* = 0.027, Figure 2D). Further, any exposure to the HFD across perinatal development increased time exhibiting stereotypy (*p* = 0.002). In contrast, inactive anxiety (Table 3) was unaffected by gender, maternal diet, or postweaning diet (*F*_1,67_ < 2.50, *p* > 0.100). Other traditional anxiety behaviors, such as crouch and freeze, were independently examined and produced no significant results (Crouch: all *p* > 0.400; Freeze: *F*_1,67_ < 2.00, *p* > 0.100).

The amount of time interacting with novel objects introduced during the behavior test decreased with postweaning HFD exposure (*F*_1,67_ = 4.874, *p* = 0.031, Figure 3A) and was lower in females (*F*_1,67_ = 5.826, *p* = 0.019, data not shown). There was an interaction between postweaning diet and gender (*F*_1,67_ = 13.613, *p* = 0.0004, Figure 3B) with postweaning HFD males interacting less with the novel objects compared to CTR males (*F*_1,67_ = 19.213, *p* = 0.00004) and compared to HFD females (*F*_1,67_ = 14.862, *p* = 0.0003). The number of interactions with novel objects was likewise decreased in postweaning HFD animals (*F*_1,66_ = 5.340, *p* = 0.024, Figure 3C). Vigilance to the novel toy and stranger was examined and found to be insignificant (*F*_1,60_ < 2.50, *p* > 0.100, data not shown). Total time spent exploring the cage was reduced in postweaning HFD offspring (*F*_1,67_ = 6.639, *p* = 0.012, Figure 3D). Examining only oral exploration of the cage showed elevation with maternal HFD exposure (percent duration: *F*_1,67_ = 5.300, *p* = 0.024, Figure 3E; total number: *F*_1,67_ = 4.350, *p* = 0.041, Figure 3F) and a reduction with postweaning HFD exposure (percent duration: *F*_1,67_ = 6.915, *p* = 0.011; total number: *F*_1,67_ = 4.415, *p* = 0.039).

There was an interaction between maternal diet and gender on time spent in locomotion (*F*_1,67_ = 6.865, *p* = 0.011, data not shown); in control offspring, females locomoted less than males (*F*_1,67_ = 4.000, *p* = 0.050). The number of locomotive behaviors was affected by an interaction between postweaning diet and gender (*F*_1,67_ = 6.047, *p* = 0.017, Figure 4B), with postweaning HFD males exhibiting fewer locomotive behaviors compared to CTR males (*F*_1,67_ = 4.135, *p* = 0.046). Postweaning CTR male offspring displayed a higher number of locomotive behaviors than females (*F*_1,67_ = 4.585, *p* = 0.036). The total number of behaviors performed during the test was affected by an interaction between postweaning diet and gender (*F*_1,67_ = 6.451, *p* = 0.013, Figure 4D). In males, postweaning HFD consumption decreased the number of behaviors exhibited during the behavior test (*F*_1,67_ = 6.588, *p* = 0.013), and within postweaning diet CTR offspring males performed more behaviors than females (*F*_1,67_ = 4.383, *p* = 0.040).

#### Maternal and Postweaning HFD Influenced Physical Activity

Activity levels during the behavior test were examined and found to decrease with postweaning HFD exposure (*F*_1,37_ = 5.327, *p* = 0.027, Figure 4E). There was an interaction with gender and maternal diet (*F*_1,37_ = 5.252, *p* = 0.028, Figure 4F), which showed that maternal HFD decreased activity in male offspring as compared to females (*F*_1,37_ = 4.406, *p* = 0.043). There was also an interaction with gender, maternal, and postweaning diet (*F*_1,37_ = 5.846, *p* = 0.021, Figure 4F). HFD/HFD males displayed reduced activity compared to females (*F*_1,37_ = 7.447, *p* = 0.010) and HFD/CTR males (*F*_1,37_ = 6.548, *p* = 0.015), but not from CTR/HFD males (*F*_1,37_ = 4.042, *p* = 0.052).

Activity level was reduced the day after the test in all offspring, regardless of diet or gender (*F*_1,31_ = 5.879, *p* = 0.021, Figure 5A). Postweaning HFD consumption increased activity independent of day examined (*F*_1,31_ = 8.701, *p* = 0.006, Figure 5A). There was an interaction between day and postweaning diet (*F*_1,31_ = 4.868, *p* = 0.035), with postweaning HFD offspring showing increased activity the day before the test (*F*_1,31_ = 15.007, *p* = 0.001). Compared to their pre-test activity levels, postweaning HFD-exposed animals exhibited decreased activity the day after the test (*F*_1,31_ = 7.327, *p* = 0.011). Furthermore, there was an interaction between day, gender, and maternal diet (*F*_1,31_ = 4.749, *p* = 0.037). After the test, maternal HFD females displayed decreased activity compared to males (*F*_1,31_ = 9.749, *p* = 0.004, Figure 5B), and from their own baseline (*F*_1,31_ = 21.016, *p* = 0.00007, Figure 5C).

#### Maternal and Postweaning HFD Influenced Cortisol

Plasma cortisol measured after the 11-month behavior test increased with postweaning HFD exposure (*F*_1,83_ = 6.729, *p* = 0.011, Figure 6A). Post-test cortisol was also affected by an interaction between maternal and postweaning diet (*F*_1,83_ = 5.772, *p* = 0.019, Figure 6A), with the HFD/HFD group increased from HFD/CTR (*F*_1,83_ = 12.966, *p* = 0.001), a postweaning diet increase, and from CTR/HFD (*F*_1,83_ = 5.489, *p* = 0.022), a maternal diet increase. Hair cortisol collected at 13 months of age was increased in males (*F*_1,49_ = 5.760, *p* = 0.020, data not shown) and unaffected by diet.

Preweaning cortisol measures were also examined for maternal diet and gender effects. Hair cortisol at weaning was increased in maternal HFD animals (*F*_1,46_ = 4.406, *p* = 0.041, Figure 6B), and plasma cortisol at weaning was increased in females (*F*_1,87_ = 3.995, *p* = 0.049, data not shown). Preweaning plasma cortisol was not found to be significant at 3 months (*F*_1,106_ < 3.00, *p* > 0.090) or 4 months of age (*F*_1,94_ < 0.100, *p* > 0.400).

#### Maternal HFD Consumption Impairs Development of the Serotonin System

**Figure 7:**
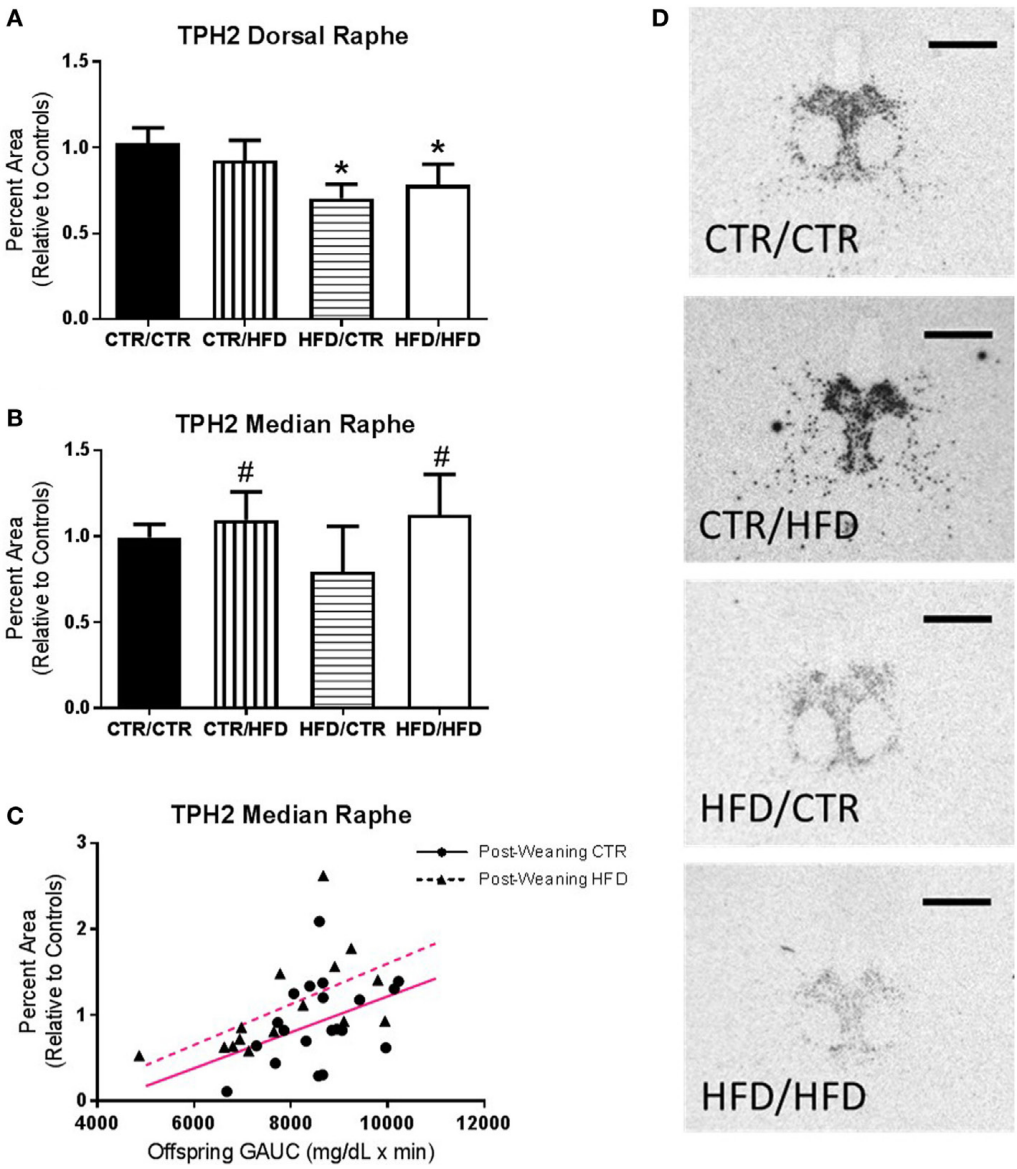
**(A)** Percent area of TPH2 mRNA expression in the dorsal raphe (DR) decreased with maternal high-fat diet (HFD) exposure (*F*_1,35_ = 5.260, *p* = 0.028). **(B)** Controlling for offspring glucose area under curve (GAUC), expression in the median raphe (MnR) increased with postweaning HFD exposure (*F*_1,26_ = 7.675, *p* = 0.010). **(C)** Offspring GAUC itself increased with MnR TPH2 mRNA expression (*F*_1,26_ = 9.719, *p* = 0.004). **(D)** Representative images of the differences seen in TPH2 mRNA Expression in the DR between the four diet groups. Data shown as mean ± SEM. * denotes maternal diet effect and ^#^ denotes a postweaning diet effect, *p* < 0.05. Magenta lines denote significant overall covariance, *p* < 0.05. Scale bars are 200 µm. Sample sizes for TPH2 mRNA expression are as follows: DR: CTR/CTR *n* = 15 (*n* = 9 males; *n* = 6 females), CTR/HFD *n* = 8 (*n* = 4 males; *n* = 4 females), HFD/CTR *n* = 8 (*n* = 5 males; *n* = 3 females), and HFD/HFD *n* = 12 (*n* = 8 males; *n* = 4 females) and MnR: CTR/CTR *n* = 13 (*n* = 7 males; *n* = 6 females), CTR/HFD *n* = 7 (*n* = 3 males; *n* = 4 females), HFD/CTR *n* = 7 (*n* = 4 males; *n* = 3 females), and HFD/HFD *n* = 8 (*n* = 5 males; *n* = 3 females).

In the DR maternal HFD exposure decreased both the percent area (*F*_1,35_ = 5.260, *p* = 0.028, Figure 7A) and density (*F*_1,35_ = 8.847, *p* = 0.005, data not shown) of TPH2 mRNA expression (Figure 7D). Expression in the MnR increased with postweaning HFD exposure (percent area: *F*_1,26_ = 7.675, *p* = 0.010, Figure 7B; Density: *F*_1,26_ = 7.172, *p* = 0.013, data not shown), as well as with increased GAUC values (percent area: *F*_1,26_ = 9.719, *p* = 0.004, Figure 7C; Density: *F*_1,26_ = 9.499, *p* = 0.005, data not shown).

The percent area of 5-HT1A mRNA expression was examined in the DR and MnR with no difference due to maternal diet, postweaning diet, or gender (all DR: *F*_1,30_ < 0.10, *p* > 0.800; all MnR: *F*_1,37_ < 3.00, *p* > 0.100). SERT expression was examined in terms of percent area and density in the DR and MnR with no significant results (all *F*_1,47_ < 2.00, *p* > 0.200).

#### Postweaning HFD Decreases 5-HT Immunoreactive Signal in the PFC

**Figure 8:**
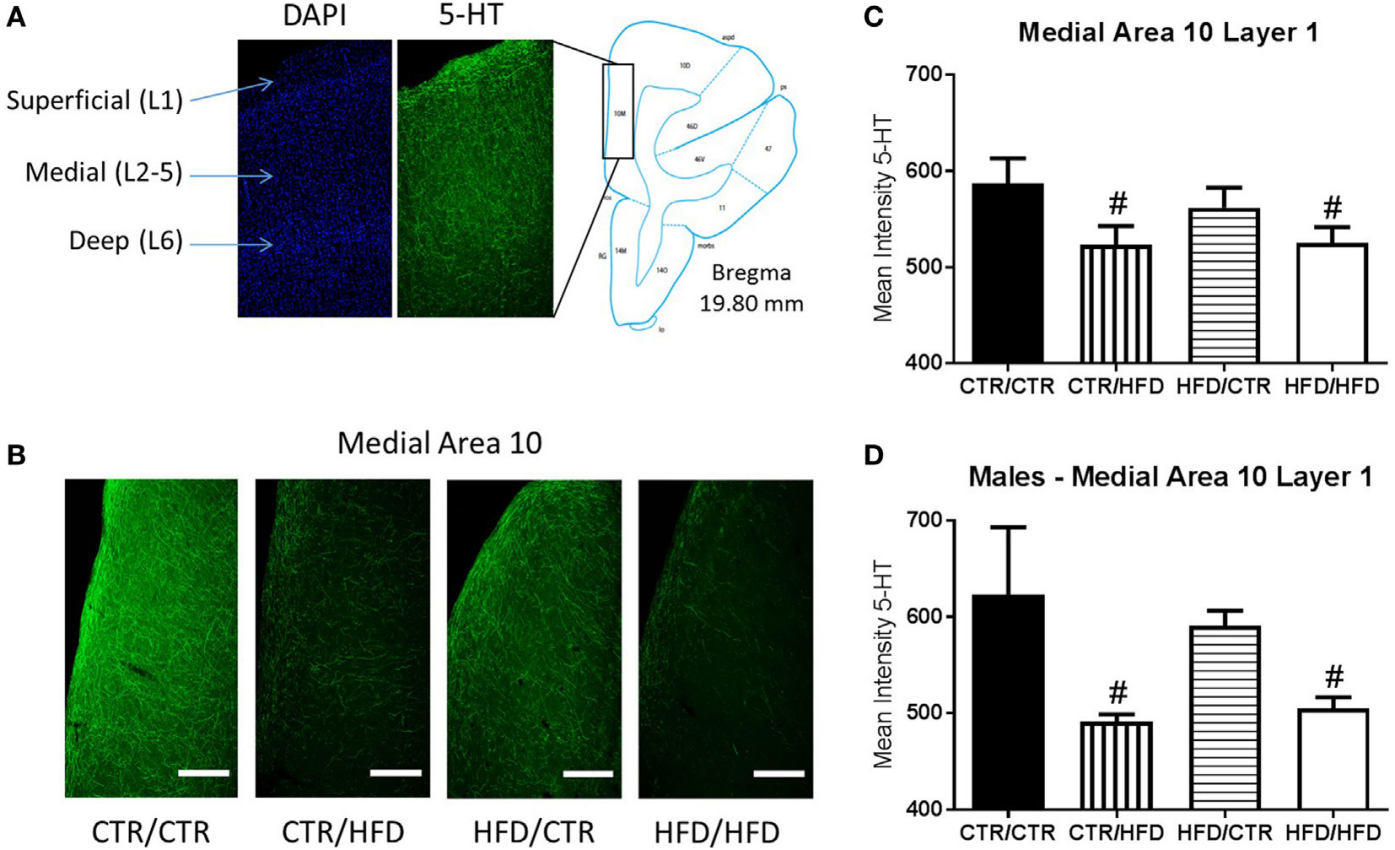
**(A)** The location of area 10 of the prefrontal cortex (PFC) was determined using figures from Paxinos Stereotaxic Atlas (39). DAPI staining was used to differentiate the layers of the cortex, numbered 1–6. **(B)** Representative images of the differences seen in 5-HT fiber innervation of medial area 10 of the PFC between the four diet groups. **(C)** Exposure to a postweaning high-fat diet (HFD) decreased mean intensity of 5-HT immunoreactive signal in layer 1 of medial area 10 in the PFC (*F*_1,16_ = 7.662, *p* = 0.014). **(D)** 5-HT immunoreactivity in the same region was further affected by an interaction between gender and postweaning diet (*F*_1,16_ = 7.497, *p* = 0.015), with mean intensity decreased in postweaning HFD males compared to control males (*F*_1,16_ = 13.895, *p* = 0.002). Data shown as mean ± SEM. ^#^ denotes a postweaning diet effect, *p* < 0.05. Scale bars are 400 µm. Sample sizes for the 5-HT immunohistochemisty are as follows: CTR/CTR *n* = 6 (*n* = 2 males; *n* = 4 females), CTR/HFD *n* = 6 (*n* = 3 males; *n* = 3 females), HFD/CTR *n* = 6 (*n* = 3 males; *n* = 3 females), and HFD/HFD *n* = 6 (*n* = 3 males; *n* = 3 females).

Intensity of 5-HT immunoreactive signal in area 10 of the medial PFC (Figure 8A) revealed an interaction between postweaning diet and gender (*F*_1,16_ = 5.712, *p* = 0.030, data not shown). Differences were more prominent in the medial region (*F*_1,16_ = 6.246, *p* = 0.024), and the effect was greatest in layer 1 (*F*_1,16_ = 6.330, *p* = 0.023, Figure 8B) in which postweaning HFD exposure alone decreased 5-HT immunoreactivity (*F*_1,16_ = 6.012, *p* = 0.026). 5-HT intensity in layer 1 of medial area 10 was decreased by postweaning HFD consumption regardless of gender (*F*_1,16_ = 7.662, *p* = 0.014, Figure 8C), and was affected by an interaction between postweaning diet and gender (*F*_1,16_ = 7.497, *p* = 0.015, Figure 8D). In males, postweaning HFD decreased mean intensity (*F*_1,16_ = 13.895, *p* = 0.002).

## Discussion

This is the first study demonstrating long-lasting effects of HFD consumption during early development on behavior and brain development in NHP offspring. The observed effects of maternal HFD appear to be due to developmental programming as the reduction in TPH2 mRNA expression in the DR and many of the behavioral aberrations persist when animals consume a healthy diet at weaning. These findings indicate that an early nutritional intervention, consumption of the control diet at weaning, was not sufficient to ameliorate many of the changes in behavior induced by maternal HFD consumption, such as increased anxiety. In addition, postweaning HFD consumption reduced serotonin immunoreactivity in area 10 of the PFC, exacerbated behavioral abnormalities, and increased stereotypy independent of maternal diet.

Results from 11-month-old male and female offspring indicate that exposure to maternal HFD increases the risk of anxiety, a risk further exacerbated by postweaning HFD consumption. This increase in anxiety in maternal HFD offspring extends and expands our previous findings of increased anxiety in 4-month-old female HFD offspring (6). It is interesting that by 11 months of age both male and female offspring now exhibit anxiety. The earlier onset of anxiety in female offspring correspond to human studies demonstrating that women are more prone to anxiety than men and that the association between obesity and anxiety disorders is more robust in women than in men (41). Importantly, the effects of maternal HFD remained significant when offspring weight was taken into account. Any exposure to a HFD during early development increased both active anxiety and stereotypy, a component of active anxiety. Total active anxiety was primarily influenced by exposure to maternal HFD, whereas stereotypy was primarily increased by postweaning HFD consumption. Stereotypy is an extreme reaction to stress exceeding the adaptive value of anxiety responses, suggesting behavioral dysregulation and reduced ability to use normative methods to alleviate anxiety (32). Our data suggest that while any developmental exposure to a HFD increases anxiety, maternal HFD is the primary determinant of anxiety displayed during behavior testing, manifesting as active forms of anxiety, and that postweaning HFD consumption independently increases stereotypy.

Postweaning HFD-exposed animals exhibited increased behavioral inhibition as evidenced by reduced interaction with novel objects and the test cage. Behavioral inhibition is associated with increased anxiety in animal models (32) and children (42, 43), as well as a lowered arousal threshold to novel stimuli (31). Postweaning HFD males displayed the highest level of behavioral inhibition, interacting the least with the novel objects. Overall, we observed that females interacted less with novel objects, a reflection of species-typical gender differences, illuminating the severity of the postweaning HFD reduction in male offspring. As no differences were observed in vigilance toward the novel objects, this decrease is not due to altered attention. Reduced interaction with the test environment provides further support that postweaning HFD consumption inhibits species-typical behavior in response to novel stimuli. Maternal HFD likewise produced a departure from normative levels of cage interaction, indicating exploratory behaviors are particularly sensitive to HFD exposure and potentially indicative of anxiety. Behavioral inhibition in children is specifically associated with social anxiety (31), suggesting that maternal and postweaning HFD exposures result in the development of varied anxiety phenotypes. Our findings that developmental HFD exposure increased anxiety are supported by several rodent studies (14, 44, 45) and recent evidence from epidemiological studies that report an association between maternal obesity and occurrence of anxiety and depression in children and adolescents (46, 47). An elevated prepregnancy BMI was associated with increased fear, sadness, and internalizing behaviors in children (8, 12). Also, maternal obesity increases the risk of abnormal birth weight (48), which in itself is associated with anxiety and depression in adolescents (13).

Postweaning HFD consumption increased baseline activity in the juveniles’ home environment as previously reported (20). We hypothesize that baseline activity is upregulated in order to defend body weight “set point” in an environment of nutritional excess. The “set point” model (49–52) proposes that circulating metabolic hormones act on the hypothalamus resulting in compensatory metabolic changes that maintain body weight at a predefined level. In contrast, postweaning HFD suppressed activity during the behavior test and in males reduced the number of behaviors performed, further indicating behavioral inhibition (53). Overall, animals exhibited reduced activity 24 h after the behavioral assessment, indicating the prolonged influence of a stressful event. Differences in the change in activity after the behavioral assessment were best explained by postweaning HFD exposure, and in females by maternal HFD exposure. The gender-specific effects of HFD/HFD exposure suggest maternal diet reprograms stress response more effectively in females, and that the postweaning diet has a greater impact on stress response in males. Thus, postweaning HFD exposure is an important regulator of physical activity, elevating baseline levels and inhibiting recovery after the stress of behavioral assessment. A variety of models, including NHPs and humans, show positive social interactions protect against deleterious effects of stress (53). The reverse effect is seen when our postweaning HFD animals return to their social group, suggesting social impairment and the experience or perception of negative social interactions (53).

Postweaning HFD consumption increased plasma cortisol response to the 11-month behavior test, a stress response further amplified with maternal HFD exposure, as HFD/HFD animals displayed the highest level of cortisol implying increased stress sensitivity. While both maternal and postweaning HFD increased acute stress response to the behavioral assessment, only males subsequently presented with an elevated chronic stress response, as measured by hair cortisol. Conversely, maternal HFD consumption increased hair cortisol at weaning. Our results are consistent with the findings from several animal models which indicate that the HPA axis is programed by perinatal HFD consumption (44, 54, 55). The impact of perinatal HFD on the HPA axis could be direct, or through increased adiposity or an increased inflammatory state induced by HFD exposure. The HPA axis is critical in behavioral regulation; in humans increased cortisol is associated with anxiety disorders (56), and NHP studies show that HPA activation and elevated plasma cortisol are associated with abnormal behaviors (57). The observed cortisol results further support our postulation that maternal and postweaning HFD exposure produce differential anxiety phenotypes. Whereas maternal HFD induces an early elevation in hair cortisol and generalized anxiety symptoms, postweaning HFD causes both an elevation in acute cortisol and social anxiety symptoms, especially in males.

We further noted that postweaning HFD exposure decreased serotoninergic immunoreactivity in area 10 of the PFC, particularly in layer 1 of the medial aspect and in males. We postulate that this reduction in serotoninergic immunoreactivity relates to decreased serotonergic innervation of area 10 of the PFC. However, differences in the density of serotonin positive fibers could also be due to differences in serotonin release and reuptake. Here too the alterations in serotonin immunoreactivity reflect the heightened sensitivity of male offspring to programming by postweaning HFD consumption. The impact of the postweaning diet on the medial PFC is not surprising, as this brain region undergoes marked growth during the juvenile period and is one of the last to fully develop (58). The reduction of serotonergic immunoreactivity in area 10 is a potential contributor to the behavioral inhibition observed in postweaning HFD animals as serotonergic innervation of the PFC is an important regulator of behavioral inhibition (59). Moreover, decreased serotonergic immunoreactivity in the PFC may underlie the observed increase in stereotypy in animals consuming the HFD postweaning, as impairments in PFC morphology are associated with increased stereotypy in a NHP model of immune activation during development (59–61). We previously demonstrated effects of maternal HFD consumption on the dopaminergic systems at 13-months of age, with maternal HFD decreasing tyrosine hydroxylase and dopamine receptor 1 and 2 protein immunoreactivity in area 10 of the PFC (35). Thus, the observed behavioral impairments could also be influenced by the dopamine system, which is modulated by serotonin activity (62).

We report that exposure to maternal HFD reduced TPH2 mRNA expression in the DR, with the programming effects of maternal HFD on TPH2 expression persisting when offspring consumed a healthy diet postweaning. In contrast, postweaning HFD consumption elevated TPH2 mRNA expression in the MnR. Similar results have been found in infant mice, with increased anxiety and depression- like behaviors resulting from decreased DR and increased MnR serotonergic activity (63). The complex projections originating from the DR and MnR are site specific and largely non-overlapping, attune to subnuclei variation (64). This divergence is apparent within cortical targets: MnR projections are concentrated in the dorsomedial components, particularly the medial PFC, and the DR projects to most cortical areas, with the medial PFC innervated more sparingly (65). The raphe nuclei also both send widespread serotonergic projections to the hypothalamus. However, the arcuate and suprachiasmatic nuclei receive input exclusively from the MnR (65). In this study, TPH2 mRNA expression was measured in the cell bodies of the DR and MnR, but not in at the axon terminals or release points along the axon where changes in 5-HT metabolism also occur. Thus, changes in TPH2 mRNA expression may indicate a reduction in the capacity to synthesize serotonin, as seen in a rodent study where inhibition of DR TPH2 mRNA expression resulted in *in vivo* suppression of serotonin synthesis (66). However, it is important to note we measured mRNA expression of the TPH2 and not the actual activity of the enzyme.

Importantly, our findings in the raphe nuclei are consistent with our observed outcomes, as the brain regions innervated by the DR and MnR are integral in the regulation of metabolism and behavior. For example, the amygdala receives robust projections from the DR (65) and increased amygdala activity indicates potential vulnerability to anxiety pathology, as the region designates learned fear response (67). Fear responses, conditioned or unpredictable, are inhibited with increased ventral PFC activity, an area key to emotional regulation (67). The medial PFC determines stressor controllability, and in humans area 10 exhibits important social functions, distinguishing between perceived and imagined stimuli (58, 68). Altered neural serotonin is associated with psychological and neurodevelopmental disorders including depression (69, 70), anxiety (71), ADHD (72), and ASD (73). Direct raphe innervation to these brain regions is implicated in the widespread serotonergic impairments seen in anxiety disorders. Serotonergic disruption is likewise involved in metabolic regulation, as serotonergic projections from the raphe nuclei synapse onto the melanocortin neurons critical in regulating energy intake and expenditure (74). In addition to the raphe nuclei, TPH2 is expressed in the hypothalamus and pituitary of humans and mice (75), and in NHPs serotonergic regulation of HPA function was shown to be TPH2-dependent (66). Unambiguously, these target areas of DR and MnR serotonergic innervation constitute a complex neural network of behavioral and metabolic regulation.

We postulate that the differential anxiety phenotypes observed in maternal and postweaning HFD groups originate from the nuclei-specific perturbations seen in the raphe serotonergic system. Exposure to a HFD during gestational and early perinatal development impairs serotonergic function of the DR neural network and results in the development of anxiety pathology. Our group has shown maternal HFD exposure suppressed serotonergic function in the fetal DR, the behavioral effects of which were seen at 4 months of age, with HFD-exposed females displaying increased anxiety (6). Hair cortisol at 6 months was elevated in male and female HFD offspring, indicating both genders experienced chronically increased stress. Intervention with a control diet postweaning had no effect on these outcomes; decreased DR TPH2 expression and increased anxiety behaviors persisted in maternal HFD offspring. Importantly, these results indicate the continued development of anxiety pathology, in spite of the remodeling capacity of the serotonergic system (76). The compounded effect of maternal and postweaning HFD on 11-month plasma cortisol and activity measures further support the long-term effects of HFD on stress response. Active anxiety was particularly increased in maternal HFD offspring, and independent of the influence of maternal HFD exposure on anxiety, low body weight was predictive of anxiety behaviors at 11 months. HFD-induced DR serotonergic deficiency could produce these effects by impairing innervation of targeted anxiety and metabolic circuits. Insufficient neuronal regulation reduces agouti-related peptide (AgRP) innervation of hypothalamic nuclei and reprograms energy balance, promoting leanness and hypophagia (77, 78). Congruent with this, our group found maternal HFD exposure reduced AgRP fibers in the paraventricular nucleus of the hypothalamus (20). Due to DR’s extensive control of behavioral and metabolic pathways, maternal HFD impairs serotonergic function and results in widespread and long-term anxiety and energy balance reprogramming.

In contrast to maternal HFD DR outcomes, postweaning HFD increased TPH2 expression, specifically in the MnR, reflecting the complexity of the development of the raphe serotonergic pathways. In depressed suicides TPH2 transcription and protein levels were increased in the DR and MnR (64, 79). Increased raphe serotonin synthesis, despite locally reduced serotonin levels, is hypothesized to compensate for insufficient serotonergic transmission at target areas (64). In area 10 of the medial PFC, one such MnR target area (65), our data show postweaning HFD exposure decreased serotonergic innervation. The introduction of a HFD during a time of elevated social stress, resulting from weaning and novel group formation, coupled with a reduced ability to exhibit control over social stressors could lead to the development of social impairment and anxiety (53). We show this to be true: at the 11-month test as postweaning HFD exposure resulted in increased behavioral inhibition, stereotypy, elevated acute stress response, and impaired ability to habituate upon return to social group. The concentration of MnR projections to area 10, coupled with the divergent effects of postweaning HFD, implicate this serotonergic pathway in the aberrant circuitry of depressive and anxiety disorders, particularly social anxiety.

The MnR’s exclusive innervation of the arcuate nucleus likewise implicates it in serotonergic regulation of metabolic pathways (65). Accordingly, we observed in this model that postweaning HFD consumption reduced AgRP fibers, this time in the arcuate nucleus (20). The increased baseline activity and hypophagia (20) seen in postweaning HFD offspring could correspond to this reduction in AgRP innervation, consistent with the body weight “set-point” hypothesis and altered energy balance. We observed a predictive effect between MnR TPH2 expression and GAUC, further evidence that postweaning HFD-induced TPH2 changes can disrupt hypothalamic metabolic homeostasis. Long-term metabolic disturbances, such as glucose intolerance and obesity, can originate from impaired function of hypothalamic neurons (77). Our findings in the MnR reflect established links between metabolic and anxiety disorders, and further explain the unique anxiety differences seen in postweaning HFD offspring.

By way of homologous but independent pathways, the MnR and DR express similar influence over frontal and HPA functions, an influence which is impaired by ontogenetic HFD exposure. The timing specific effects of HFD exposure on the direction and location of TPH2 expression support our results indicating maternal and postweaning HFD consumption generate unique behavioral and physiological changes. The incorporation of gender as a risk factor for anxiety further explains our results (80). HFD females display anxiety earlier than males, and are more impacted by the compounded effects of maternal and postweaning HFD exposure. Males appear to be selectively affected by postweaning HFD exposure, with chronic suppression of HPA function and decreased serotonin immunoreactive fibers in the PFC. Importantly, any developmental HFD exposure causes long-term aberrations in the serotonergic system, with risk factors such as exposure period and gender contributing to differential pathology presentation.

Our findings in the central serotonin system suggest neural mechanisms for differential anxiety development in HFD-exposed NHP offspring, and provide evidence that childhood diet impacts neural development. While other factors in our model, such as maternal obesity and hyperinsulinemia, may also influence offspring development, chronic HFD exposure is the primary mediator of the observed impairments in behavior and brain development. This hypothesis was generated based on previous findings from our group demonstrating that, independent of maternal adiposity, increasing maternal dietary fat contributes to elevated offspring percent body fat, inflammatory markers, and stress activation (19, 21). Investigation of the later postnatal period reaffirmed these results as postnatal HFD consumption had no effect on weight gain or metabolic rate (20), and still increased inflammatory response. In combination with our results of maternal and postweaning HFD exposure altering neural development independent of juvenile metabolic phenotype, evidence strongly suggests that the diet—not resulting metabolic phenotype—is the primary source of mechanistic control.

We postulate that the observed HFD-induced behavioral and serotonergic impairments are due to increased exposure to pro-inflammatory factors. In our NHP model, we previously documented increased circulating and hypothalamic cytokines in fetal HFD offspring at the third trimester (16). Neural development is susceptible to the deleterious effects of inflammatory stress, particularly the serotonin system. Serotonergic neurons are sensitive to inflammatory events, as elevation of inflammatory cytokines in rats reduced the survival of embryonic serotonin neurons in the rostral raphe (81) and resulted in degeneration of serotonergic axons in the amygdala and PFC (82). NHP offspring prenatally exposed to pro-inflammatory Immunoglobulin G class antibodies from mothers of children with ASD displayed increased anxiety responses such as stereotypy and hyperactivity, indicative of serotonergic dysfunction (83). In humans elevated levels of inflammatory cytokines in obese pregnant women are associated with increased risk of anxiety, depression, and ASD (84). As the brainstem raphe nuclei are the main center for serotonin production, they are likely key to these perturbations.

In conclusion, we demonstrated that HFD consumption during early development has long-lasting effects on NHP offspring behavior and brain development. Maternal HFD changes appear to be due to developmental programming as the behavioral and serotonergic pathologies produced are unaffected by early dietary intervention. Female offspring are particularly prone to maternal HFD effects. Postweaning HFD consumption was found to exacerbate behavioral inhibition and increase stereotypy, especially in males. Future studies will address the relative importance of maternal diet and obesity in offspring development, and investigate the impact of specific dietary components. Changes in early postnatal environmental factors including maternal mental health and maternal-infant interactions may explain some of the observed behavioral and neuroendocrinological dysfunctions. Further studies will directly examine the impact of developmental HFD exposure on offspring social behavior and cognition. Given the high prevalence of HFD consumption and obesity in developed countries, and the potential these factors have to increase the risk of developing neuropsychiatric and neurodevelopmental disorders, it is crucial that future studies identify efficacious therapeutic interventions.

## Ethics Statement

All animal procedures were in accordance with National Institutes of Health guidelines on the ethical use of animals and were approved by the Oregon National Primate Research Center (ONPRC) Institutional Animal Care and Use Committee.

## Author Contributions

ES conceived the project and designed the research; JT, JV, AB, JF, MD, JB, and ES performed the experiments; JT and ES analyzed the data; all authors discussed the data; JT, JV, and ES wrote the manuscript with contributions from all authors.

## Conflict of Interest Statement

The authors declare that the research was conducted in the absence of any commercial or financial relationships that could be construed as a potential conflict of interest.
